# Normative values for the video Head Impulse Test in children without otoneurologic symptoms and their evolution across childhood by gender

**DOI:** 10.1007/s00405-023-07900-6

**Published:** 2023-03-09

**Authors:** Rosana Rodríguez-Villalba, Miguel Caballero-Borrego

**Affiliations:** 1 Department of Otorhinolaryngology, Hospital Sant Joan de Déu, Barcelona, Spain; 2 Department of Otorhinolaryngology, Althaia Xarxa Assistencial Universitària de Manresa, Manresa, Spain; 3 Otorhinolaryngology-Head and Neck Surgery Department, Hospital Clínic, University of Barcelona, C/Villarroel, 170, Esc. 8, 2ª, 08036 Barcelona, Spain; 4 Institut d’Investigacions Biomèdiques Agusti Pi Sunyer (IDIBAPS), University of Barcelona, Barcelona, Spain

**Keywords:** Adolescent, Balance, Children, Vertigo, Vestibulo-ocular reflex, Video head impulse test

## Abstract

**Purpose:**

The video Head Impulse Test is routinely used to assess semicircular canal function in adults, but to date, pediatric reference values are scarce. This study aimed to explore the vestibulo-ocular reflex (VOR) in healthy children at different development stages and to compare the obtained gain values with reference to those in an adult population.

**Methods:**

This prospective, single-center study recruited 187 children from among patients without otoneurological diseases, healthy relatives of these patients, and staff families from a tertiary hospital. Patients were divided into three groups by age: 3–6 years, 7–10 years, and 11–16 years. The vestibulo-ocular reflex was assessed by video Head Impulse Test, using a device with a high-speed infrared camera and accelerometer (EyeSeeCam^®^; Interacoustics, Denmark).

**Results:**

We found a lower vestibulo-ocular reflex gain of both horizontal canals in the 3–6-year-old group when compared with the other age groups. No increasing trend was found in the horizontal canals from age 7–10 years to age 11–16 years, and no differences were found by sex.

**Conclusion:**

Gain values in the horizontal canals increased with age until children reached age 7–10 years and matched the normal values for adults.

## Introduction

Vertigo is a more common complaint among adults than in the pediatric population [1]. However, the incidence of vestibular disorders during childhood is rising, probably due to greater awareness of their existence and the improvements in otoneurological and vestibular diagnosis [2–5]. Indeed, various reports describe children with vestibular dysfunction [6–10], including those presenting as complications after cochlear implantation [11], Meniere’s disease [12], otitis media with effusion [13], cytomegalovirus infection [14], primary ciliary dyskinesia [15], sensorineural hearing loss [16], and migraine [8, 17]. In response, criteria have recently been published for the diagnosis and management of vestibular migraine of childhood, probable vestibular migraine of childhood, and recurrent vertigo of childhood [18], based on patient-reported clinical features. Otoneurological evaluation and the diagnosis of vestibular disorders are also more challenging in young children than in adults because of the difficulty describing unsteadiness and vertigo accurately [17, 19, 20]. Consequently, vestibular disorders are often confused with neurological, motor, or coordination problems, leading to other specialists assessing them before referral to an otolaryngologist, which could delay the achievement of motor skills [21]. All patients with these symptoms should therefore undergo objective tests of their vestibular function [22].

The examination of eye movement is of utmost importance for the diagnosis of vestibular disorders and should include assessment of the vestibulo-ocular reflex (VOR), nystagmus, and saccades [23]. Most vestibular tests evaluate the VOR, which acts to stabilize the gaze during head movements. Nystagmus refers to physiological or pathological involuntary eye movement, with pathology typically resulting from central nervous system disorders, toxicity, alcohol, and drugs. *Saccades* consist of a rapid eye movement that orientates gaze toward a target and locate its image onto the fovea [17, 24]. However, the VOR may only be altered during or shortly after an episode of vertigo, with disturbances occurring when asymptomatic [25]. Vestibular diagnosis is difficult in young children because it requires collaboration and can be influenced by anatomical and functional artifacts [26, 27].

The video Head Impulse Test (vHIT) is typically used to record abnormal eye movements or saccades, which may not be visible to the naked eye, and provide quantitative assessment of the VOR [26, 27]. This can reveal vestibular dysfunction. Furthermore, the vHIT can quantify semicircular canal function by measuring VOR gain (the ratio of the eye movement response to passive head movement applied during acceleration) [28]. To complete the test and minimize errors caused by slippage, patients must wear tight-fitting goggles that hold a camera. This tight fit can be difficult for children to tolerate and is a major reason for artifacts in the pediatric population [22, 29]. The vHIT should also be performed with high rotational velocities (150°/s or more) because most patients with unilateral vestibular dysfunction will have normal VOR gains at lower speeds [20, 30]. It is possible, for example, that lower velocities are experienced during normal activities that require compensatory processes. Therefore, pulses at low velocities should be considered invalid and discarded.

Another problem with the VOR gain recorded by the vHIT is the lack of normal values for the pediatric populations. Whereas some consensus exists for normal values in adults [30, 31], the lack of data on vHIT values in children without dizziness forces a reliance on adult reference values irrespective of age. Therefore, this study aimed to evaluate the VOR gain values measured by vHIT in children without otoneurological symptoms and to quantify their evolution through childhood. These results may be useful as reference values when evaluating children with vestibular disorders.

## Methods

### Subjects

This prospective study included 187 children from among healthy patients, the healthy relatives of those patients, and the families of staff at our tertiary hospital. Patients were divided into three age groups (3–6, 7–10, and 11–16 years) after excluding those diagnosed with otoneurologic or ophthalmologic disorders, sleep apnea, and morbid obesity, as well as those using vestibular sedatives and wearing corrective glasses. A minimum sample size of 30 subjects per group was required to achieve statistical power.

Written informed consent was provided by all parents. The study was conducted in accordance with the ethical standards of the Declaration of Helsinki and was approved by the Ethics committee of our institution (file number: PS-09-20).

### Protocol

All subjects underwent complete otomicroscopic and otoneurologic examination, including tympanometry, Dix-Hallpike and McClure maneuvers, and balance assessment by the Romberg, tandem walk, and Unterberger–Fukuda tests. We then performed the vHIT between February 2019 and March 2021 to assess the VOR, using a device with a high-speed infrared camera and accelerometer (EyeSeeCam^®^ Interacoustics, Denmark). This provided data on the gain values for the six semicircular canals, together with the presence or absence of saccades. Children were asked to fix their sight on a dot placed on the wall 1 m away, with cartoon stickers used to attract the attention of children aged 3–6 years old. All data were collected by the same examiner. Tests considered invalid due to a lack of co-operation, excessive blinking, involuntary cervical muscle contraction, or any other factor that diminished reproducibility were discarded. Consistent with the protocol reported by Zamaro et al., artifacts or invalid impulses due to high or low test velocity were also excluded [32].

### Statistical analysis

Categorical data were compared using chi-squared or Fisher’s exact tests, as appropriate, and continuous data were assessed using analysis of variance. The Mann–Whitney *U* test was used to compare the mean VOR gain values by age and gender, with Bonferroni multiple comparison post-hoc tests used to compare median VOR gain values among the age groups. The relationship between the VOR gain values in any canal and age was analyzed by Spearman’s rank correlation. All statistical analyses were performed using IBM SPSS, version 25.0 (IBM Corp., Armonk, NY, USA), and *P*-values of < 0.05 were considered statistically significant.

## Results

**Fig. 1:**
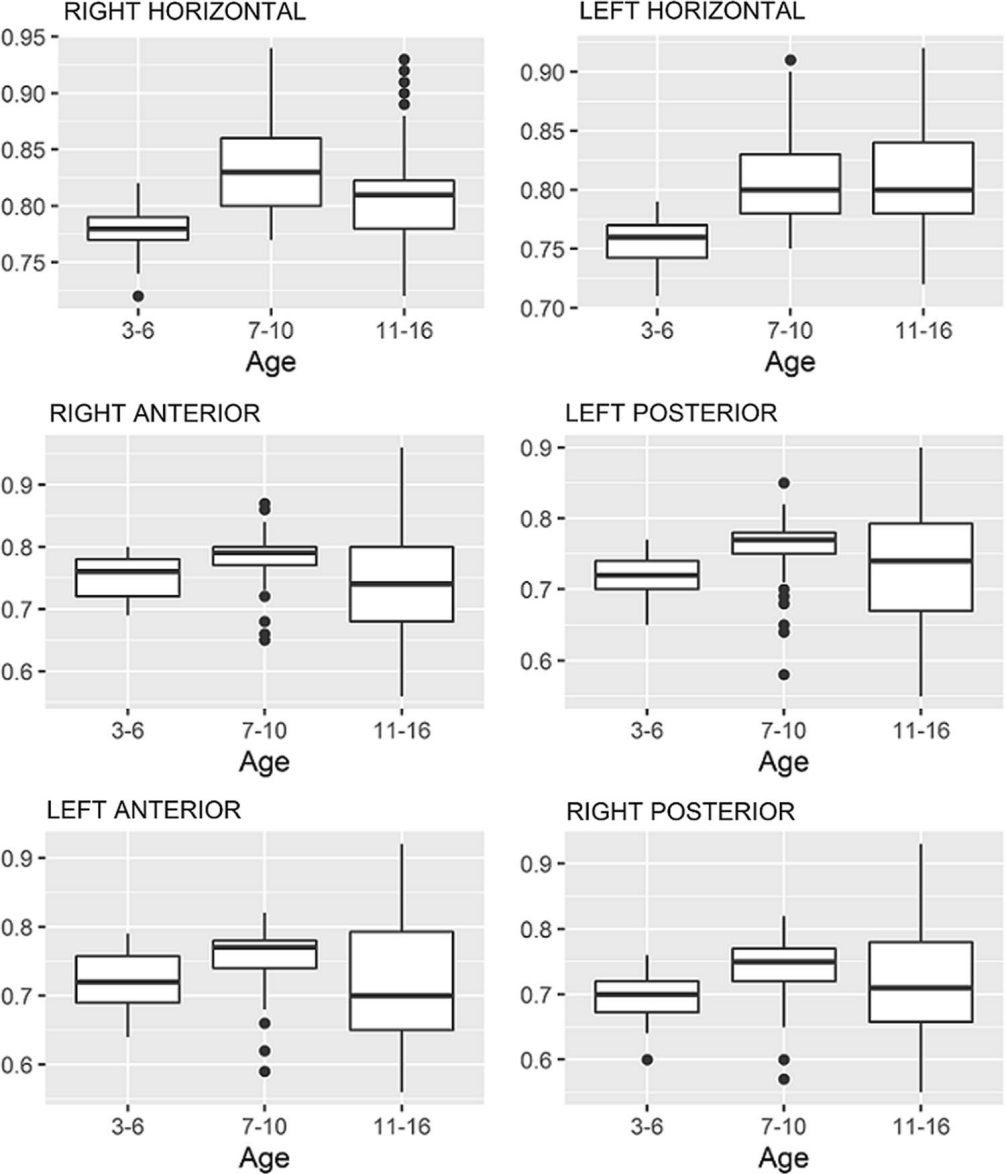
Box chart of the gain values for the vestibulo-ocular reflex in the six canals by age

**Fig. 2:**
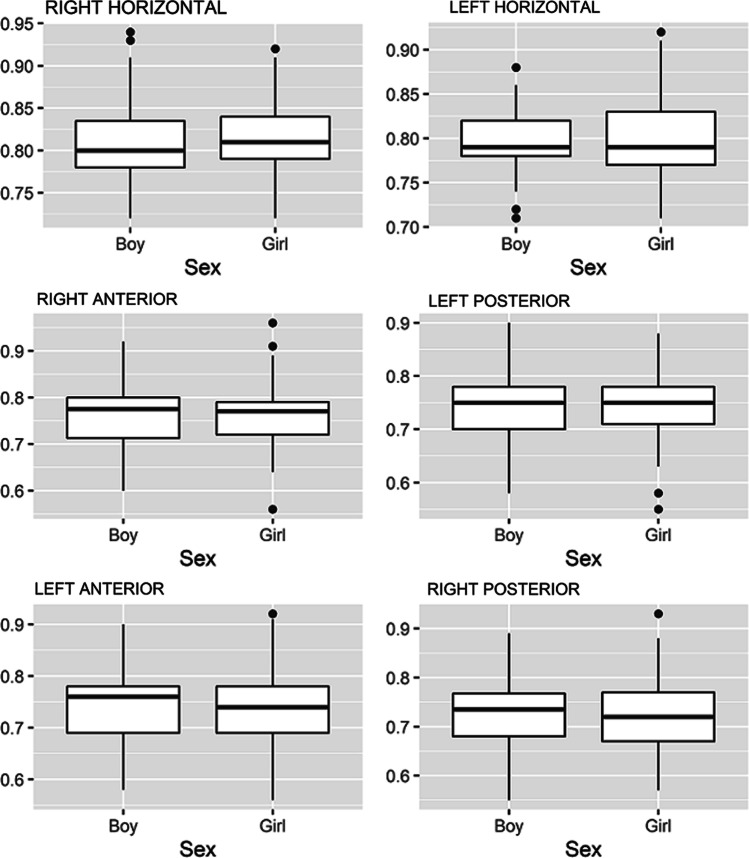
Box chart of the gain values for the vestibulo-ocular reflex in the six canals by gender

Of the 187 children (117 girls and 70 boys) enrolled in this study, 34 (18.2%) were aged 3–6 years old, 69 (36.9%) were aged 7–10 years old, and 84 (44.9%) were aged 11–16 years old. Table 1 and Fig. 1 show the VOR gains for the six semicircular canals by age. The mean VOR gains differed between the three age groups in all six canals (*P* < 0.001). Bonferroni’s multiple comparison post-hoc tests (Table 2) revealed that the VOR gain values in 3–6-year-old group were most relevant to daily clinical practice, with lower results than in the other groups for both horizontal canals. No increasing trends were observed in the horizontal canals from the 7–10-year-old to 11–16-year-old groups. Spearman’s rho correlation of the mean gains in each canal by age detected a positive correlation in both horizontal canals, though this was lower in the right (0.157) than in the left (0.384) (Table 3). No correlation was demonstrated in the anterior or posterior canals. Finally, Table 4 and Fig. 2 show the VOR gain values by sex, revealing no statistical differences (*P* > 0.001) in the values for any canal between the two groups.

**Table 1:** Vestibulo-ocular reflex gain values in each semicircular canal by age

Semicircular canal	Age															*P*
	3–6 Years Old					7–10 Years Old					11–16 Years Old					
	Mean	SD	Median	P25	P75	Mean	SD	Median	P25	P75	Mean	SD	Median	P25	P75	
Right horizontal	0.78	0.02	0.78	0.77	0.79	0.83	0.04	0.83	0.80	0.86	0.82	0.05	0.81	0.78	0.83	< 0.001
Left horizontal	0.76	0.02	0.76	0.74	0.77	0.81	0.04	0.80	0.78	0.83	0.81	0.04	0.80	0.78	0.84	< 0.001
Right anterior	0.75	0.03	0.76	0.72	0.78	0.78	0.04	0.79	0.77	0.80	0.75	0.08	0.74	0.68	0.80	< 0.001
Left posterior	0.72	0.03	0.72	0.70	0.74	0.76	0.04	0.77	0.75	0.78	0.74	0.08	0.74	0.67	0.80	< 0.001
Left anterior	0.72	0.04	0.72	0.69	0.76	0.76	0.04	0.77	0.74	0.78	0.72	0.09	0.70	0.65	0.80	< 0.001
Right posterior	0.70	0.04	0.70	0.67	0.72	0.74	0.04	0.75	0.72	0.77	0.71	0.08	0.71	0.66	0.78	< 0.001

**Table 2:** Bonferroni’s multiple comparison post-hoc tests of vestibulo-ocular reflex gain values in each semicircular canal by age

Semicircular canal	Age group (years)		
	3–6 vs 7–10	3–6 vs 11–16	7–10 vs 11–16
Right horizontal	** < 0.001**	** < 0.001**	**0.013**
Left horizontal	** < 0.001**	** < 0.001**	1.000
Right anterior	**0.002**	1.000	**0.001**
Left posterior	** < 0.001**	**0.040**	0.069
Left anterior	**0.004**	1.000	**0.001**
Right posterior	**0.001**	0.335	**0.013**

Bold values denote statistical significance

**Table 3:** Spearman’s rho correlation of mean gains with age for each semicircular canal. Bold values denote statistical significance.

Semicircular canal	Correlation coefficient	*P*
Right horizontal	0.157	**0.032**
Left horizontal	0.384	** < 0.001**
Right anterior	−0.051	0.488
Left posterior	0.107	0.144
Left anterior	−0.059	0.421
Right posterior	0.025	0.736

Bold values denote statistical significance

**Table 4:** Vestibulo-ocular reflex gain values for each semicircular canal by sex

Semicircular canal	Participant sex										*P*
	Girl					Boy					
	Mean	SD	Median	P25	P75	Mean	SD	Median	P25	P75	
Right horizontal	0.82	0.04	0.81	0.79	0.84	0.81	0.05	0.80	0.78	0.84	0.245
Left horizontal	0.80	0.04	0.79	0.77	0.83	0.80	0.04	0.79	0.78	0.82	0.639
Right anterior	0.76	0.06	0.77	0.72	0.79	0.76	0.06	0.78	0.71	0.80	0.494
Left posterior	0.74	0.06	0.75	0.71	0.78	0.74	0.06	0.75	0.70	0.78	0.973
Left anterior	0.73	0.07	0.74	0.69	0.78	0.74	0.08	0.76	0.69	0.78	0.699
Right posterior	0.72	0.06	0.72	0.67	0.77	0.72	0.07	0.74	0.68	0.77	0.591

## Discussion

Evidence suggests that the incidence of vestibular disorders is increasing in pediatric populations [3, 4, 8–10, 33, 34]. Although vHIT is among the most widely used methods for studying vertigo, normal VOR gain values have not been established for young children. This may reflect the difficulty in performing a complete vestibular evaluation, especially in the young and when patients may have hearing loss, neurological disorders, or developmental disorders [1, 5, 26].

The present study reports the vHIT results for healthy children at three developmental stages to provide reference values. We show that the VOR gains of the horizontal semicircular canals were more homogeneous than those of the anterior or posterior canals. This probably reflects the relative ease of applying head acceleration to study the horizontal semicircular canals compared with the vertical canals [29], even though most children have better ranges of neck movement than older people. Although Wiener–Vacher and Wiener have also reported normal values for infants and children, their results are not comparable to ours because they used a remote camera system to avoid intolerance of the tight-fitting goggles in classical vHIT testing [29]. Some authors also describe that children produce more artifacts than adults and that greater care is required to avoid technical errors in children [26].

Cut-off values for VOR gain may vary by age even in adult populations, though normal values probably sit between 0.8 and 1.0 [27]. The researchers also demonstrated an optimal cut-off point of 0.7 (specificity, 100%; sensitivity, 67%) in a non-healthy pediatric cohort [27], consistent with our results among younger children. It seems that the VOR gain is low in children younger than 3 years old [20] and that it follows a rapid increase until age 6 years. Therefore, progress slows to age 16 years, when adult values are achieved [20, 29], and finally decrease again from age 80 years [30, 31]. We observed a similar evolution, with the trend for VOR gains in the horizontal canals to increase rapidly until age 7–10 years and then stabilize to age 11–16 years old. Most studies also confirm that no differences exist between older children or adolescents and adults [27, 35, 36]. Contrasting with these results, however, some authors have not observed changes from age 4 years to age 18 or 20 years [27, 37]. Some of the variation among these findings may result from the different devices used, as demonstrated by van Dooren et al. when comparing the EyeSeeCam (Interacoustics VOG; Munich, Germany), ICS Impulse (GN Otometrics; Taastrup, Denmark), and Ulmer (Synapsys, Marseille, France) in adults [38]. For example, when analyzing normative values for semicircular canal function in adolescents aged 11–18 years with the Otometrics IC Impulse vHIT, Emekci et al. obtained mean VOR gain values higher than ours (i.e., 0.89 and 0.87 for the lateral and posterior canals, respectively) [39]. They also found no age-related differences among adolescents, consistent with our data for adolescents aged 11–16 years old [39]. When using the EyeSeeCam device, Retamal et al. found no differences between their 5–10-year-old and 11–17-year-old groups, although this research was also limited by a particularly small sample size [40]. Surprisingly, and contrasting with our findings, they also found differences between the VOR gain values of the right and left horizontal canals [40].

Although the vestibular end organs are considered mature even before birth [41], anatomical and functional development could explain the changes in VOR gain with age. Notably, central pathways in the cerebellum that are important for controlling the VOR lack maturation at birth and mature during the first years of life [42]. Another reason for could be that anatomical changes occur in eye size and interpupillary distance during childhood [43]. Children under 3 years of age often have hypermetropia, which could compromise their view of the target during the vHIT [44]. These factors, together with the lack of co-operation and poor fit of goggles among young children [27], justify the exclusion of children aged < 3 years from our study.

The main limitations of this study are the difficulty in performing examinations in young children and the differences in anatomical development and neurological maturation between subjects in each age group. Both factors may have affected the results. Future prospective studies should include more participants grouped into shorter age groups to define the range of normal test values for children at different ages.

## Conclusion

The vHIT is a reliable, non-invasive, and easy-to-perform test that can be used in emergency rooms to assess children with dizziness or vertigo, potentially avoiding the need for costly CT scans or MRI that frequently require sedation. However, the vHIT test results are difficult to interpret in pediatric populations because standard gain values have not been fully established for children without otoneurological pathology. Our results suggest that the gain values of horizontal canals increase with age until age 7–10 years, when the normal values remain largely stable to adolescence and approximate normal values in adults. We also found no differences by sex. The reference values identified in this study have the potential to inform the evaluation of children with vestibular disorders. Research with other vestibular tests should now try to elucidate the evolution or maturation of pathways necessary for the VOR and how these affect age-specific normal values.

## Data Availability

The data that support the findings of this study are available on request from the corresponding author, [MC], upon reasonable request.

## References

[1] Maudoux A, Vitry S, El-Amraoui A (2022). Vestibular deficits in deafness: clinical presentation, animal modeling, and treatment solutions. Front Neurol.

[2] Christy JB (2018). Considerations for testing and treating children with central vestibular impairments. Semin Hear.

[3] Brodsky JR, Lipson S, Bhattacharyya N (2020). Prevalence of pediatric dizziness and imbalance in the United States. Otolaryngol neck Surg Off J Am Acad Otolaryngol Neck Surg.

[4] Hülse R, Biesdorf A, Hörmann K (2019). Peripheral vestibular disorders: an epidemiologic survey in 70 million individuals. Otol Neurotol.

[5] Hazen M, Cushing SL (2021). Vestibular evaluation and management of children with sensorineural hearing loss. Otolaryngol Clin North Am.

[6] Dhondt C, Dhooge I, Maes L (2019). Vestibular assessment in the pediatric population. Laryngoscope.

[7] Zhou G, Goutos C, Lipson S, Brodsky J (2018). Clinical significance of spontaneous nystagmus in pediatric patients. Int J Pediatr Otorhinolaryngol.

[8] Devaraja K (2018). Vertigo in children; a narrative review of the various causes and their management. Int J Pediatr Otorhinolaryngol.

[9] Wiener-Vacher SR, Quarez J, Le PA (2018). Epidemiology of vestibular impairments in a pediatric population. Semin Hear.

[10] Sommerfleck PA, González Macchi ME, Weinschelbaum R (2016). Balance disorders in childhood: main etiologies according to age. Usefulness of the video head impulse test. Int J Pediatr Otorhinolaryngol.

[11] Jacot E, Van Den Abbeele T, Debre HR, Wiener-Vacher SR (2009). Vestibular impairments pre- and post-cochlear implant in children. Int J Pediatr Otorhinolaryngol.

[12] Abouzari M, Abiri A, Djalilian HR (2019). Successful treatment of a child with definite Meniere’s disease with the migraine regimen. Am J Otolaryngol Head Neck Med Surg.

[13] Tozar M, Cömert E, Şencan Z (2020). Video head impulse test in children with otitis media with effusion and dizziness. Int J Pediatr Otorhinolaryngol.

[14] Dhondt C, Maes L, Oostra A, Dhooge I (2019). Episodic vestibular symptoms in children with a congenital cytomegalovirus infection: a case series. Otol Neurotol.

[15] Zawawi F, Papsin BC, Dell S, Cushing SL (2022). Vestibular and balance impairment is common in children with primary ciliary dyskinesia. Otol Neurotol.

[16] Gadsbøll E, Erbs AW, Hougaard DD (2022). Prevalence of abnormal vestibular responses in children with sensorineural hearing loss. Eur Arch Otorhinolaryngol.

[17] Rodríguez-Villalba R, Caballero-Borrego M, Villarraga V (2020). Vestibulo-ocular reflex assessed with video head impulse test in children with vestibular migraine: our experience. Int J Pediatr Otorhinolaryngol.

[18] Van De Berg R, Widdershoven J, Bisdorff A (2021). Vestibular migraine of childhood and recurrent vertigo of childhood: diagnostic criteria consensus document of the committee for the classification of vestibular disorders of the Bárány Society and the international headache society. J Vestib Res Equilib Orient.

[19] Gruber M, Cohen-Kerem R, Kaminer M, Shupak A (2012). Vertigo in children and adolescents: characteristics and outcome. Sci World J.

[20] Janky KL, Rodriguez AI (2018). Quantitative vestibular function testing in the pediatric population. Semin Hear.

[21] Singh A, Raynor EM, Lee JW (2021). Vestibular dysfunction and gross motor milestone acquisition in children with hearing loss: a systematic review. Otolaryngol Neck Surg.

[22] Hülse R, Hörmann K, Servais JJ (2015). Clinical experience with video head impulse test in children. Int J Pediatr Otorhinolaryngol.

[23] Kheradmand A, Zee DS (2012). The bedside examination of the vestibulo-ocular reflex (VOR): an update. Rev Neurol (Paris).

[24] Beraneck M, Lambert FM, Sadeghi SG, Romand R, Varela-Nieto I (2014). Functional development of the vestibular system: sensorimotor pathways for stabilization of gaze and posture. Development of auditory and vestibular systems.

[25] Bernetti L, Pellegrino C, Corbelli I (2018). Subclinical vestibular dysfunction in migraineurs without vertigo: a clinical study. Acta Neurol Scand.

[26] Kim K-S, Jung YK, Hyun KJ (2020). Usefulness and practical insights of the pediatric video head impulse test. Int J Pediatr Otorhinolaryngol.

[27] Hamilton SS, Zhou G, Brodsky JR (2015). Video head impulse testing (VHIT) in the pediatric population. Int J Pediatr Otorhinolaryngol.

[28] Yang CJ, Lee JY, Kang BC (2016). Quantitative analysis of gains and catch-up saccades of video-head-impulse testing by age in normal subjects. Clin Otolaryngol.

[29] Wiener-Vacher SR, Wiener SI (2017). Video head impulse tests with a remote camera system: normative values of semicircular canal vestibulo-ocular reflex gain in infants and children. Front Neurol.

[30] McGarvie LA, MacDougall HG, Halmagyi GM (2015). The video head impulse test (vHIT) of semicircular canal function—age-dependent normative values of VOR gain in healthy subjects. Front Neurol.

[31] Strupp M, Grimberg J, Teufel J (2020). Worldwide survey on laboratory testing of vestibular function. Neurol Clin Pract.

[32] Zamaro E, Tehrani ASS, Kattah JC (2020). VOR gain calculation methods in video head impulse recordings. J Vestib Res Equilib Orient.

[33] Balzanelli C, Spataro D, Redaelli de Zinis LO (2021). Prevalence of pediatric and adolescent balance disorders: Analysis of a mono-institutional series of 472 patients. Children.

[34] Fancello V, Palma S, Monzani D (2021). Vertigo and dizziness in children: an update. Children.

[35] Janky KL, Givens D (2016). Vestibular, visual acuity and balance outcomes in children with cochlear implants: a preliminary report. Ear Hear.

[36] Ross LM, Helminski JO (2016). Test–retest and interrater reliability of the video head impulse test in the pediatric population. Otol Neurotol.

[37] Lehnen N, Ramaioli C, Todd NS (2017). Clinical and video head impulses: a simple bedside test in children. J Neurol.

[38] van Dooren TS, Starkov D, Lucieer FMP (2020). Comparison of three video head impulse test systems for the diagnosis of bilateral vestibulopathy. J Neurol.

[39] Emekci T, Uğur KŞ, Cengiz DU, Men Kılınç F (2021). Normative values for semicircular canal function with the video head impulse test (vHIT) in healthy adolescents. Acta Otolaryngol.

[40] Retamal SR, Díaz PO, Fernández AM (2021). Assessment protocol and reference values of vestibulo-ocular reflex (VOR) gain in the horizontal plane recorded with video-head impulse test (VHIT) in a pediatric population. Codas.

[41] Nikolaeva EI, Efimova VL, Vergunov EG (2022). Integration of vestibular and auditory information in ontogenesis. Children.

[42] Romero JE, Coupe P, Lanuza E (2021). Toward a unified analysis of cerebellum maturation and aging across the entire lifespan: a MRI analysis. Hum Brain Mapp.

[43] Helo A, Pannasch S, Sirri L, Rämä P (2014). The maturation of eye movement behavior: scene viewing characteristics in children and adults. Vision Res.

[44] Mezer E, Meyer E, Wygnansi-Jaffe T (2015). The long-term outcome of the refractive error in children with hypermetropia. Graefe’s Arch Clin Exp Ophthalmol Albr von Graefes Arch fur Klin und Exp Ophthalmol.

